# Electrostatically driven fluorescent sensor for rapid detection of AChE activity and organophosphate pesticides via dual-enzyme cascade amplification

**DOI:** 10.3389/fphar.2025.1679948

**Published:** 2025-11-28

**Authors:** Xiaoyi Liu, Kunhui Sun, Sitong Lai, Guojing Liu, Meifang Li, Ping Wang, Shuhong Wang, Lan Ma, Xie-An Yu, Wei Lei, Bing Wang

**Affiliations:** 1 Key Laboratory of Pharmacology of Traditional Chinese Medical Formulae, Ministry of Education, Tianjin University of Traditional Chinese Medicine, Tianjin, China; 2 Shenzhen Institute for Drug Control, Shenzhen, China; 3 Tsinghua Shenzhen International Graduate School, Shenzhen, China

**Keywords:** Azo-Bodipy 685, fluorescent sensor, acetylcholinesterase, organophosphorus pesticides, electrostatic self-assembly

## Abstract

**Introduction:**

The widespread use of organophosphorus pesticides (OPs) in Chinese herbal medicines cultivation raises urgent concerns about residue contamination. Conventional detection methods [e.g., gas chromatography-mass spectrometry (GC-MS) and enzyme-linked immunosorbent assay (ELISA)] suffer from poor portability and instability of antibody inactivation in complex matrices, hindering on-site analysis.

**Methods:**

This study proposed a novel “electrostatic adsorption-driven cascade reaction chain” strategy for rapid detection of acetylcholinesterase (AChE) activity and OPs. Leveraging the electrostatic self-assembly between a positively charged acetylcholine chloride (ACh, 26.47 ± 1.63 mV) and a negatively charged choline oxidase (CHO, −30.81 ± 1.85 mV), a nanoscale fluorescence sensor (CA-B NPs) was constructed by encapsulating the Azo-Bodipy 685. This design created a spatially confined and componentially co-localized nanoreactor that restricted substrate diffusion distance to the nanoscale and utilized a dual-enzyme cascade system (AChE-CHO) to yield a signal amplification effect.

**Results:**

The obtained CA-B NPs exhibited excellent analytical performance, including: (1) a low detection limit of 4.1 ng/mL for triazophos; (2) high recovery of 88.13%−113.09% in complex Citrus reticulata Blanco matrices, along with strong anti-interference capabilities by organically dividing the reaction and detection sections; (3) a total assay time of only 20 min for real samples, suitable for rapid, on-site, high-throughput screening.

**Discussion:**

This study not only embedded the entire reaction chain [AChE-CHO-hydrogen peroxide (H_2_O_2_)] into the sensor to improve space utilization efficiency and detection efficiency, but also established a novel paradigm for enzyme spatial organization based on electrostatic complementarity, providing new insights into the rational design of nanostructured multi-enzyme sensing platforms.

## 1 Introduction

Organophosphorus pesticides (OPs), accounting for 40% of global pesticide usage, are widely applied in agriculture (Zhao et al., 2020a) and Chinese herbal medicines cultivation (Zheng et al., 2022; Zhao et al., 2024; Zhang B. et al., 2025). These residues may accumulate in organisms through food chains, causing neurotoxicity (e.g., dizziness, nausea) or even fatal respiratory failure (Sule et al., 2022; Xu et al., 2023; Bai et al., 2025). To safeguard people’s health, it is essential to determine whether the residual level of OPs in traditional Chinese herbal medicines are above the threshold. Numerous analytical techniques have been developed for the detection of OPs, including chromatographic techniques such as gas chromatography-mass spectrometry (GC-MS) (Zhang et al., 2016), liquid chromatography-mass spectrometry (LC-MS) (Shim et al., 2022), immunoassays like enzyme-linked immunosorbent assay (ELISA) (Zhou et al., 2022; Xu et al., 2022), lateral flow immunoassay (LFIA) (Gao et al., 2019), and capillary electrophoresis (CE) (Chen et al., 2021). Unfortunately, the inherent limitations of these conventional techniques significantly hinder their application in the rapid detection of pesticide residues. For instance, GC-MS and LC-MS are highly sensitive for routine analysis of OPs residues, however, their dependence on complex instrumentation renders them inadequate for on-site screening requirements (Mohd Kamal et al., 2022; Chen et al., 2017; Shurubor et al., 2017). Immunoassays are constrained by antibody denaturation under harsh assay conditions, limiting their widespread applicability (Gong et al., 2022; Woo et al., 2013). Moreover, the complexity of these techniques (Zhao et al., 2020b; Zhao et al., 2018), combined with the need for highly trained personnel, further reduces their accessibility and efficiency in field-deployable pesticide screening. These challenges collectively underscore the urgent need to develop novel analytical approaches that are not only rapid, but also simple, cost-effective, and suitable for on-site application.

**SCHEME 1:**
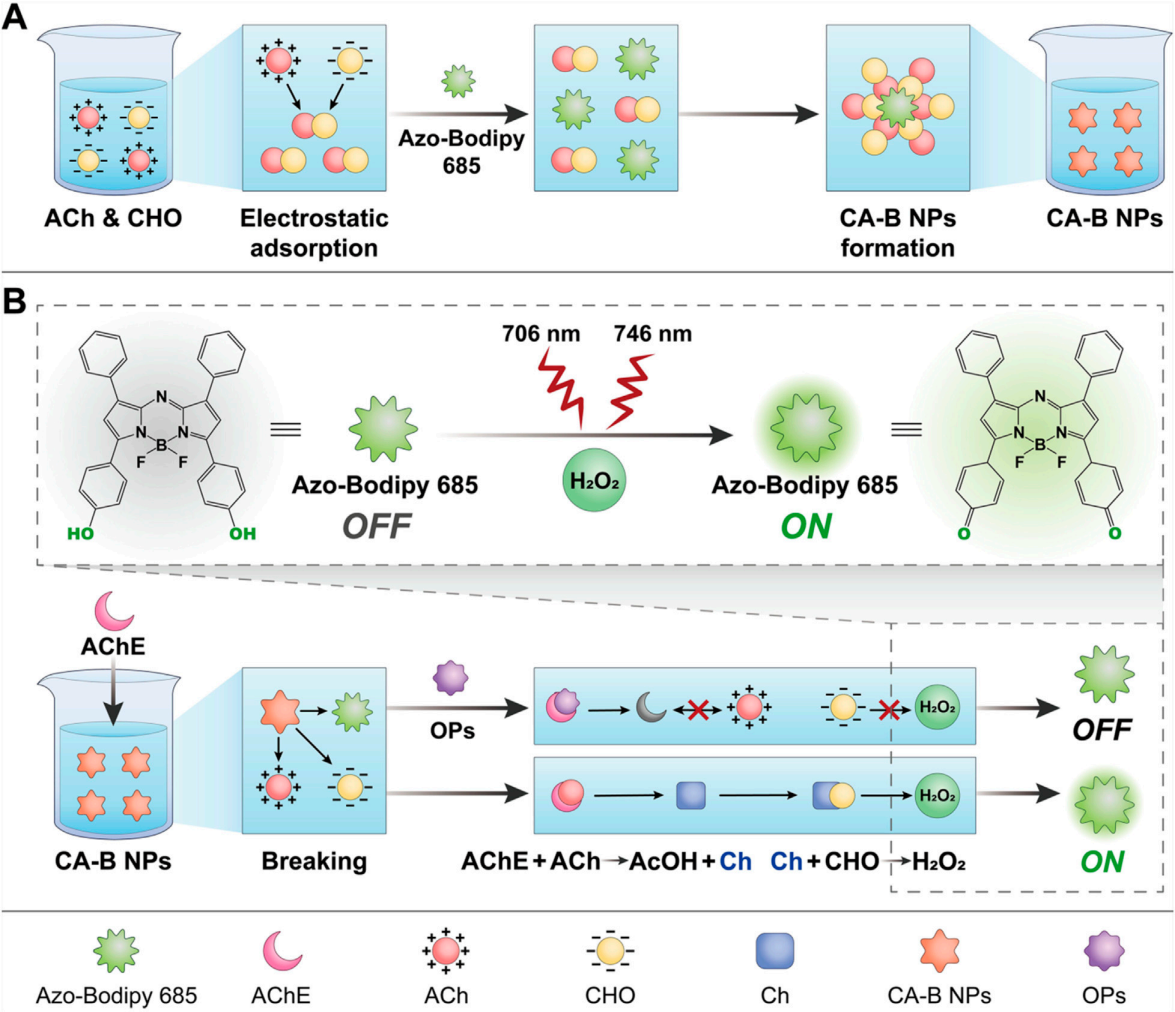
Schematic illustration of the electrostatic adsorption-driven fluorescent sensor CA-B NPs for detection. **(A)** Synthetic route of CA-B NPs; **(B)** Principle of fluorescence-based detection of AChE activity using CA-B NPs and enzyme inhibition-based detection of OPs residues.

Currently, fluorescence-based methods are attracting attention as promising alternatives for the detection of OPs due to their rapid response (Wu et al., 2023), operational simplicity, high sensitivity and compatibility with high-throughput screening (Chang et al., 2022). Recent advances continue to underscore this potential, with the development of novel platforms such as nanozyme-based deep learning systems for multi-OPs differentiation, and ratiometric fluorescent probes that offer improved anti-interference capability (Li et al., 2025; Zhang L. et al., 2025). However, the core challenge of fluorescence technology for OPs detection lies in optimising the trade-off between detection efficiency, cost-effectiveness and analytical performance. To date, most acetylcholinesterase (AChE)-targeted fluorescent probes have achieved specific molecular recognition and high signal transduction (Satnam et al., 2018; Kumar et al., 2016). Furthermore, most fluorescent sensing systems rely on indirect detection of AChE catalytic secondary products to reflect enzyme activity and OPs content, with the considerable preparation cost and diverse signal transduction of such indirect product-based detection systems. Nonetheless, their practical applications remain limited by several intrinsic drawbacks, the heterogeneity of the materials leads to low output efficiency and hinders the analysis of complex samples (Zhao et al., 2020a). Fundamentally, most fluorescent methods cause the following problems due to the introduction of heterogeneous components: (1) diffusion constraints: Brownian dynamics simulations reveal that excessive interlayer spacing in 2D nanostructures significantly extends diffusion distances and reduces the collision frequency of AChE (Xiao et al., 2021); (2) steric hindrance: Immobilized choline oxidase (CHO) on nanoparticle surfaces may suffer from substantial activity loss due to active-site blockage (Ahlawat et al., 2023); (3) matrix interference: Complex sample environments, such as herbal or food matrices, further compromise detection accuracy. To address these challenges, we developed an “electrostatic adsorption-driven cascade reaction chain” strategy, leveraging the electrostatic complementarity between acetylcholine chloride (ACh) and CHO. It was shown that AChE catalyzes the hydrolysis of a positively charged substrate ACh, and its hydrolysis product choline chloride, which was catalyzed by negatively charged CHO to generate hydrogen peroxide (H_2_O_2_) (Wu et al., 2021; Korram et al., 2020; Huang et al., 2019; Zhao F. et al., 2023). Therefore, the fluorescent probe was fused with the sensor system and embedded into the complete reaction chain (AChE-CHO-H_2_O_2_) using the charge complementarity-driven self-assembly technology to form an integrated sensing design, achieving a charge-directed, spatially confined reaction environment. The enzyme-substrate spatial distribution can be optimised by electrostatic adsorption, which was expected to facilitate substrate diffusion and reduce steric hindrance (Yang et al., 2024), avoid the interference of heterogeneous components, and enhance the detection efficiency and specificity, so as to realise a rapid detection of the activity of AChE and the content of OPs in the complex sample matrices.

Herein, we proposed an “electrostatic adsorption-immobilised probe and dual-enzyme cascade” strategy to construct a novel condensation-driven fluorescent sensor. In this system, CHO and ACh were electrostatically self-assembled into Choline Oxidase-Acetylcholine chloride NanoParticles (CA NPs), encapsulating the fluorescent probe Azo-Bodipy 685 to form the CA-B NPs (Scheme 1). This architecture possessed two key functionalities: (1) nanoscale spatial confinement: Through electrostatic adsorption between oppositely charged ACh (Zeta = 26.47 ± 1.63 mV) and CHO (Zeta = −30.81 ± 1.85 mV), a highly confined nano-reactive microenvironment was established on the surface of Azo-Bodipy 685, significantly reducing the diffusion distance of enzymatic substrates; (2) dual-enzyme cascade signal amplification: AChE hydrolyzed ACh to generate choline chloride, which was subsequently catalysed by CHO to produce H_2_O_2_, thus triggering a fluorescence response of the encapsulated probe. Specifically, the nanoconfined interfacial architecture elevated the local concentration of choline chloride, which was the hydrolysis product of AChE-ACh interaction, thereby accelerating CHO-mediated catalytic cycling and generating H_2_O_2_, which further triggered the response of the fluorescent probe and amplified the fluorescent signal. In the presence of OPs, AChE activity was inhibited, resulting in reduced choline chloride production and consequently diminished the amount of H_2_O_2_ catalytically produced by CHO, leading to a weakened fluorescence signal. Importantly, CA-B NPs demonstrated high responsiveness to AChE activity and enabled rapid detection of OPs. This cascade reaction enabled a amplification (one molecule of AChE catalytic event contributing to the production of H_2_O_2_), not only improving the utilisation of response space, but also lowering the detection limit through cascade amplification. The sensor exhibited a limit of detection (LOD) as low as 0.17 U/L for AChE and 4.1 ng/mL for the triazophos at an emission wavelength of 746 nm. In some aspects superior to other sensing strategies, including portable devices and highly sensitive systems utilizing metal-organic frameworks (Xiao et al., 2025; Yan et al., 2023; Zhao Y. et al., 2023). In addition, the detection time was 20 min for the analysis of real samples, which provided convenience for high-throughput screening on site. This design establishes a cascade paradigm characterized by reaction-diffusion synergy, bridging the gap between enzyme kinetics and nanoscale spatial confinement, and lays the foundation for future development of multi-target detection platforms.

## 2 Materials and methods

### 2.1 Reagents and equipment

Azo-Bodipy 685 was provided by Xi’an Ruixi Biotechnology Co., Ltd. (Xi’an, China). Acetylcholinesterase (AChE) (210 U/g, 100 mg), choline oxidase (CHO) (100 U) and acetylcholine chloride (ACh) (500 mg) were purchased from MCE Co., Ltd. (Shanghai, China). Triazophos solution (100 μg/mL, 1 mL) and chlorpyrifos solution (100 μg/mL, 1 mL) were acquired from Tianjin Alta Science and Technology Co., Ltd. (Tianjin, China). Dimethyl sulfoxide (DMSO) was purchased from Shanghai Macklin Biochemical Co., Ltd. (Shanghai, China). Acetonitrile (ACN) and methanol (MeOH), both of which are chromatographic grade, were obtained from Merck Co., Ltd. (Darmstadt, Germany). Hydrogen peroxide (H_2_O_2_) (content: 30%) was provided by Guangzhou Chemical Reagent Factory Co., Ltd. (Guangzhou, China). 1M Tris-HCl buffer (PH = 8.0, 500 mL) was purchased from Wuhan Pumeike Biology technology Co., Ltd. (Hubei, China). The water used in the experiment was deionized and ultrafiltration by Milli-Q IQ 7000 device.

Nanoparticle morphology was assessed using a Talos transmission electron microscope (TEM) (Thermo Fisher, Talos F200X G2, Czech Republic). A dynamic light scattering device (Malvern Panalytical Instruments, Zetasizer Nano 100 90, England) was used to measure the hydrodynamic size and zeta potential at room temperature. Fluorescence and ultraviolet-visible (UV-Vis) absorbance data were proceeded using a microplate reader (Thermo Scientific Varioskan Flash, 3001, Finland). Enzymes were incubated for viability assays using a biochemical incubator at 37 °C (Binder, KB53, Germany). The fluorescent imaging were recorded on the IVIS Lumina Series III (PerkinElmer, USA) with excitation at 706 nm and emission at 746 nm. Stability tests under different environmental conditions were conducted using constant temperature and humidity chamber (Memmert, HPP750, Germany). And an X-ray photoelectron spectroscopy (XPS) was detected using a spectrometer (Thermo Scientific, K-Alpha, USA).

### 2.2 Synthesis of CA-B NPs

CHO-ACh nanoparticles (CA NPs) were synthesized via an electrostatic co-assembly strategy. To obtain the optimal process parameters, the synthesis conditions were optimized by using the control variable method. Specifically, 20 μL of CHO solution (0.2 U/μL) and 80 μL of ACh solution (50 mg/mL) were added sequentially to 900 μL of Tris-HCl buffer solution (pH = 8). The mixture was stirred in the dark at 1,000 rpm for 2.5 h under sealed conditions. Upon completion of the reaction, the resulting CA NPs were collected and stored in a refrigerator at 4 °C protected from light for subsequent use.

The procedure for the synthesis of CA NPs was used as a template for the further synthesis of CHO-ACh-Azo-Bodipy nanoparticles (CA-B NPs). Firstly, 60 μL of Azo-Bodipy 685 fluorescent probe (1 mg/mL in ACN) was slowly added dropwise into 900 μL of Tris-HCl buffer solution, during which the solution was stirred at 1,000 rpm for 1 h to yield fluorescent nanoparticles containing the probe of Azo-Bodipy 685 (B NPs). Subsequently, CHO (20 μL, 0.2 U/μL) and ACh (80 μL, 50 mg/mL) solution were introduced into the system and stirred for an additional 2.5 h. Upon completion of the reaction, the mixture was allowed to stand overnight in the dark to allow complete evaporation of ACN, affording the final CA-B NPs and stored in a refrigerator protected from light at 4 °C for subsequent use.

### 2.3 Characterization of CA-B NPs

The physicochemical properties of the nanoparticles, including morphology, hydrodynamic size, zeta potential, XPS, UV-Vis absorption and fluorescence spectra, were systematically characterized. The morphology of CA-B NPs, CA NPs and B NPs were tested using TEM with an accelerating voltage of 200 KV. All the nanoparticles were performed for dynamic light scattering and zeta potential using a Malvern Panalytical Instrument at room temperature. In addition, UV-Vis data as well as fluorescence data including their fluorescence spectra at the excitation wavelength of 706 nm were further recorded by a microplate reader, which facilitated further visual fluorescence imaging using a fluorescent imaging system. CA-B NPs sample were performed and analyzed through the XPS with power.

### 2.4 Performance verification of sensor response to AChE

In this study, a stepwise and hierarchical validation strategy was followed, based on the logical framework of “monomer components, electrostatic self-assembly and all-in-one encapsulation” to assess the responsiveness to AChE:

Firstly, the enzyme cascade amplification was verified at the level of monomer component. AChE solution (5 μL, 0.01 U/μL) was placed in the Eppendorf (EP) tube containing 40 μL Tris-HCl buffer solution and incubated at 37 °C for 5 min. Subsequently, ACh solution (4.5 μL, 50 mg/mL) was added and incubated at 37 °C for 10 min. Then, CHO solution (0.5 μL, 0.2 U/μL) was introduced and further incubated at 37 °C for 10 min. Finally, 50 μL of Azo-Bodipy 685 dissolved in DMSO was added to terminate the enzymatic reaction and further detected the fluorescence signals (λex/em = 706/746 nm).

Next, enzyme cascade amplification was verified at the level of CA NPs formation by electrostatic adsorption assembly. AChE solution (5 μL, 0.01 U/μL) was placed into the EP tube and incubated at 37 °C for 5 min. Subsequently, 40 μL of CA NPs were continued to be added, mixed thoroughly, and then continued to be incubated for another 20 min at 37 °C, and finally, 50 μL of DMSO solution containing Azo-Bodipy 685 was added to terminate the enzyme reaction and to further detect fluorescence signals (λex/em = 706/746 nm).

Finally, enzyme cascade amplification was verified at the level of electrostatically complexed immobilised probes forming CA-B NPs. The AChE solution was pre-incubated at 37 °C for 5 min, followed by the addition of 40 μL CA-B NPs. After thorough mixing, the mixture was incubated in the dark at 37 °C for an additional 20 min. The reaction was then terminated by adding 50 μL of DMSO, and the intensity of fluorescence signals were recorded using a microplate reader (λex/em = 706/746 nm).

### 2.5 Standard curve detection of AChE activity using CA-B NPs and CA NPs

Different concentrations of AChE solutions (10 μL) were placed into EP tubes and incubated at 37 °C for 5 min. Subsequently, 40 μL of CA NPs were continued to be added, mixed thoroughly, and then continued to be incubated at 37 °C for another 20 min. Finally, 50 μL of DMSO solution containing Azo-Bodipy 685 was added to terminate the enzyme reaction, and the fluorescence signals were measured (λex/em = 706/746 nm).

Similarly, following the above procedure, the AChE solution was first incubated at 37 °C for 5 min, followed by the addition of 40 μL CA-B NPs, mixed well, and then placed in a biochemical incubator at 37 °C to avoid light for another 20 min. And finally 50 μL DMSO was added to terminate the enzyme cascade reaction system to continue to use the microplate reader to measure and record the intensity of fluorescence signals (λex/em = 706/746 nm).

The final concentrations of AChE were 0, 0.5, 1, 5, 10, 25, 50, 100, 200, 300, 400, 500 and 1000 U/L. The fluorescence increase rate (%) was calculated using the formula: (F–F_0_)/F_0_, where F_0_ was the fluorescence intensity measured in the absence of AChE, and F was the fluorescence intensity measured in the presence of AChE. All the above experiments were repeated three times for the measurements. In addition, the detection limit was calculated using the equation: 3σ/k, where σ was the standard deviation of repeated value of the blank response and k was the slope of the calibration curve.

### 2.6 Standard curve detection of triazophos and chlorpyrifos using CA-B NPs

To establish the standard curve for pesticide detection, 5 μL of triazophos methanol solution at various concentrations and 5 μL of AChE solution (0.01 U/μL) were added into the EP tubes and incubated at 37 °C for 20 min. Then, 40 μL of CA-B NPs was added, mixed thoroughly, and the mixture was incubated in the dark at 37 °C for another 20 min. Subsequently, 50 μL of DMSO was added to terminate the enzyme cascade reaction, and to continue to collect the intensity of fluorescence signals (λex/em = 706/746 nm) to construct the calibration curve.

Following the same procedure, standard curves were also constructed for chlorpyrifos in acetonitrile solution (5 μL) at various concentrations.

The concentration gradients of triazophos in the above system were 0, 0.0075, 0.01, 0.05, 0.075, 0.1, 0.5, 0.75 and 1 μg/mL, and those of chlorpyrifos were 0, 0.03, 0.05, 0.07, 0.1, 0.3, 0.5, 0.7 and 1 μg/mL. Fluorescence intensity was recorded using a microplate reader with an excitation wavelength of 706 nm and emission at 746 nm. All measurements were performed in triplicate.

The inhibition rate (%) was calculated using the formula: (F–F_1_)/F, where F was the fluorescence intensity of CA-B NPs in the presence of AChE but without OPs, and F_1_ was the fluorescence intensity of CA-B NPs in the presence of both AChE and the OPs.

### 2.7 Detection of triazophos in real samples

Sample extraction solutions containing triazophos at concentrations of 0.05, 0.1 and 0.5 μg/mL were prepared by spiking the pesticide into *Citrus reticulata* Blanco (Chenpi) extracts. For each concentration, 5 μL of the sample extract was mixed with 5 μL of AChE solution (0.01 U/μL) and incubated at 37 °C for 20 min. Then, 40 μL of CA-B NPs was added, and the mixture was further incubated for 20 min at 37 °C. Finally, 50 μL of DMSO was added to terminate the reaction, followed by fluorescence measurements. Each experiment was performed in triplicate for validation.

Fifteen batches of authentic Chenpi samples were selected, with batch numbers: S2410050, S2409650, S2407290, S2406550, S2406090, S2405170, S2404720, S2404830, S2404160, S2403480, S2403340, S2403100, S2402270, S2402010 and S2402020. Pesticide residues in these samples were extracted following the sample preparation method specified in the Chinese Pharmacopoeia (2022 Edition) for pesticide residue determination. The above detection procedure was applied to all 15 batches to evaluate the presence of OPs residues.

### 2.8 Statistical analysis

The obtained data were all statistically analyzed using Origin 2018. Data are represented as mean ± standard deviation (SD). The test was used to test the data statistics between two groups, and Dunnett’s test was used after one-way ANOVA when there were three or more groups. *P < 0.05 is considered to have a statistical difference. **P < 0.01 is considered to have significant statistical difference. ***P < 0.001 is considered to have extremely significant statistical difference.

## 3 Results and discussion

### 3.1 Photophysical properties of Azo-Bodipy 685

Boron-dipyrromethene (Bodipy) is a highly efficient fluorophore with excellent photophysical properties, including a high molar extinction coefficient, high fluorescence quantum yield, and outstanding photostability (Liu et al., 2022). Moreover, its emission wavelength and solubility can be tuned through the introduction of various substituents (Gomez and Lopez, 2019). Among them, Azo-Bodipy 685, a Bodipy-based fluorescent probe containing two hydroxyl groups, was employed in this study for the detection of AChE activity and pesticide residues (Supplementary Figure S1).

**FIGURE 1:**
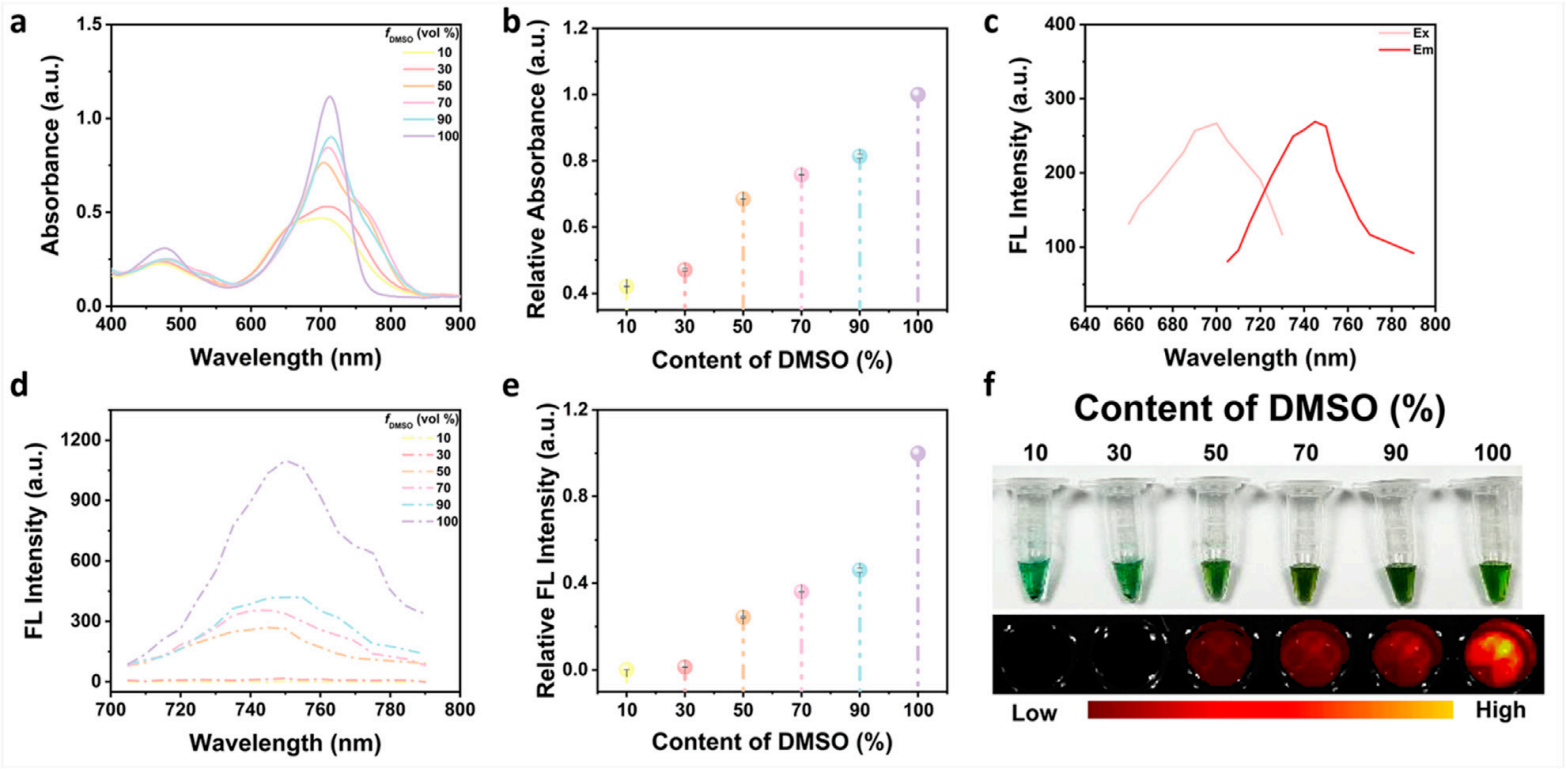
Photophysical properties of Azo-Bodipy 685 in DMSO/Tris-HCl mixtures with different DMSO volume fractions (f_DMSO_). **(a)** UV-Vis absorption spectra; **(b)** Maximum UV-Vis absorption intensity; **(c)** Optimal excitation and emission wavelengths of Azo-Bodipy 685 in 50% DMSO solution; **(d)** Fluorescence spectra; **(e)** Maximum fluorescence intensity; **(f)** Bright field and corresponding fluorescence imaging. All error bars represent standard deviations based on three parallel measurements.

As shown in the UV-Vis absorption spectra of Azo-Bodipy 685 (Figure 1a), the amphiphilic structure of the molecule led to spontaneous formation of ordered aggregates in aqueous solution. Consequently, the absorbance intensity of the maximum absorption peak of Azo-Bodipy 685 decreased with decreasing DMSO content in the solvent (Figure 1b), accompanied by the occurrence of a redshift. Notably, in a solvent containing 50% DMSO, a more distinct absorption was observed compared to that in 30% DMSO. Furthermore, as shown in Figure 1c, the optimal excitation and emission wavelengths of Azo-Bodipy 685 were 706 nm and 746 nm, respectively. As depicted in Figures 1d,e, the fluorescence intensity of Azo-Bodipy 685 increased with the rising DMSO content, with a significant enhancement observed in the 50% DMSO solution compared to the 30% DMSO condition. Moreover, the aggregation behavior of Azo-Bodipy 685 in mixed DMSO/Tris-HCl solvents of different ratios was intuitively visualised (Figure 1f). Aggregation produced by Azo-Bodipy 685 increased with higher Tris-HCl content, resulting in a reduction in fluorescence, while started to produce pronounced fluorescence in 50% DMSO solvent. Based on these findings, the solution containing 50% DMSO was selected as the optimal solvent condition for subsequent experiments. Immediately after that, the reaction properties of B NPs were further explored, finding that its fluorescence intensity increased with the concentration of H_2_O_2_ in a linear relationship (Y = 0.0605X + 1.1021, R^2^ = 0.99) (Supplementary Figure S2), demonstrating its H_2_O_2_-responsiveness and laying the foundation for the integration of the probe into the enzyme cascade reaction system.

### 3.2 Synthesis and characterization of CA-B NPs

**FIGURE 2:**
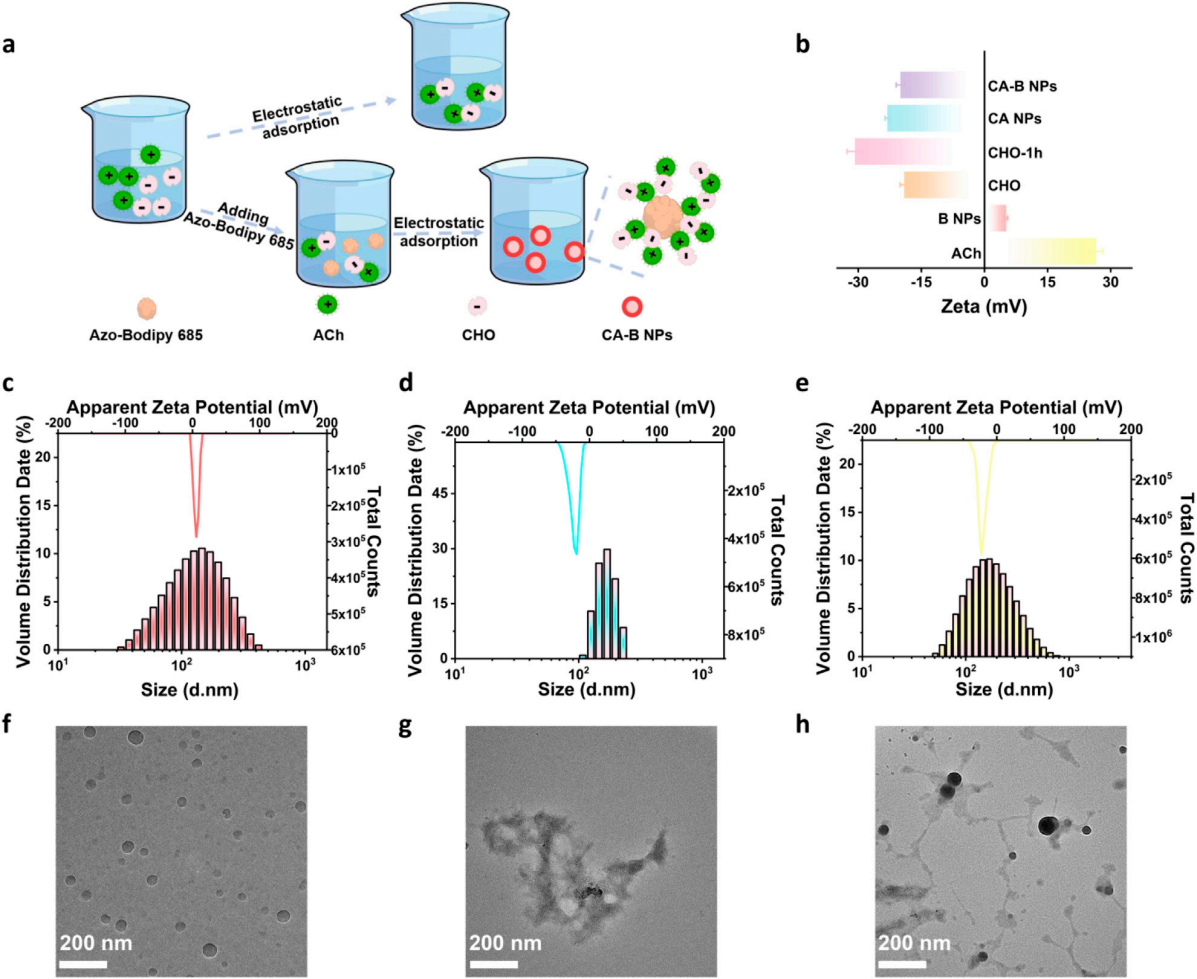
Characterization of CA-B NPs. **(a)** Synthesis diagram of CA-B NPs by electrostatic adsorption of ACh/CHO onto Azo-Bodipy 685-loaded nanoparticles; **(b)** Zeta potential changes of individual components, CA NPs and CA-B NPs; Hydrodynamic diameter and zeta potential of **(c)** B NPs, **(d)** CA NPs and **(e)** CA-B NPs; TEM images of **(f)** B NPs, **(g)** CA NPs and **(h)** CA-B NPs. All error bars represent standard deviations based on three parallel measurements.

The synthetic route of CA-B NPs was illustrated in Figure 2a. Specifically, CHO and ACh were first assembled via electrostatic interaction and coalesced to form CA NPs. Using these CA NPs as templates, B NPs were subsequently encapsulated to form CA-B NPs. In this process, the synthesis parameters were optimized using a single-variable control approach, focusing on the ratio of biorecognition elements and different stirring reaction time. The optimal preparation conditions for CA-B NPs were determined as follows: a CHO:ACh ratio of 4 U:5 μmol (Supplementary Figures S3a, b), a stirring reaction time of 1.5 h for component ACh in CA NPs formation (Supplementary Figures S3c, d), and a 1 h stirring period for encapsulating Azo-Bodipy 685 into CA-B NPs (Supplementary Figures S3e, f).

During preparation under optimal conditions, the changes of zeta potential in solution provided evidence for nanoparticle formation (Figure 2b). Specifically, stirring CHO alone in Tris-HCl buffer (pH = 8) at room temperature for 1 h resulted in a zeta potential change from −19.10 ± 0.96 mV (Figure 2d) to −30.81 ± 1.85 mV. Upon the addition of ACh (initial Zeta potential: 26.47 ± 1.63 mV) and further stirring for 1 h, CA NPs were formed with a zeta potential of −23.11 ± 0.51 mV, and the change in zeta potential of the solution (ΔZeta = 7.70 ± 0.51 mV) indicated that CHO and ACh drove the coalescence to form CA NPs via electrostatic adsorption. Encapsulation of B NPs (Zeta potential: 5.13 ± 0.37 mV, Figure 2c) into the CA NPs yielded CA-B NPs with a final zeta potential of −19.98 ± 1.00 mV (Figure 2e), resulting in a ΔZeta potential of 3.13 ± 1.00 mV. This significant change indicated the successful formation of CA-B NPs. Furthermore, the absolute value exceeding 18 mV indicated good stability of the CA-B NPs.

Subsequently, dynamic light scattering (DLS) analysis showed the average hydrodynamic diameter of CA-B NPs to be approximately 169.50 ± 2.79 nm (Figure 2e), comparable to 150.98 ± 2.67 nm for CA NPs (Figure 2d) and 118.86 ± 0.31 nm for B NPs (Figure 2c). TEM provided visual confirmation of morphology: B NPs appeared nearly spherical (Figure 2f), CA NPs exhibited fibrous structures (Figure 2g), and the final morphology of CA-B NPs displayed fibrous matrices encapsulating spherical nanoparticles (Figure 2h). XPS result was given in Supplementary Figure S4a. The chemical compositions of CA-B NPs were detected, including C1s (284.99 eV), N1s (398.83 eV), and O1s (531.19 eV). It was found that the binding energy peaks of C1s (Supplementary Figures S4b) were super imposed including 284.80 eV, 286.41 eV, 288.02 eV and 289.34 eV, which were related to the C-C/C-H bond, C-O/C-N bond, C=O/C=N bond and O-C=O bond of the CHO, ACh and B NPs structure, respectively. The high resolution O1s spectrum showed the occurrence of C=O functional group at 531.43 eV, and C-O functional group bond at 532.53 eV in CA-B NPs (Supplementary Figure S4c). High resolution N1s spectrum could be separated into peaks at 398.66 eV, 399.87 eV and 401.27 eV, attributed to the attendance of O=C-N bond, C-N bond and NR_4_ (R = C/H) bond, respectively (Supplementary Figure S4d). Together, the zeta potential shifts, DLS results and TEM images confirmed that CHO and ACh successfully self-assembled via electrostatic interaction to encapsulate B NPs, resulting in the formation of stable CA-B NPs.

### 3.3 Verification of fluorescence response mediated by enzyme cascade reaction in CA-B NPs

**FIGURE 3:**
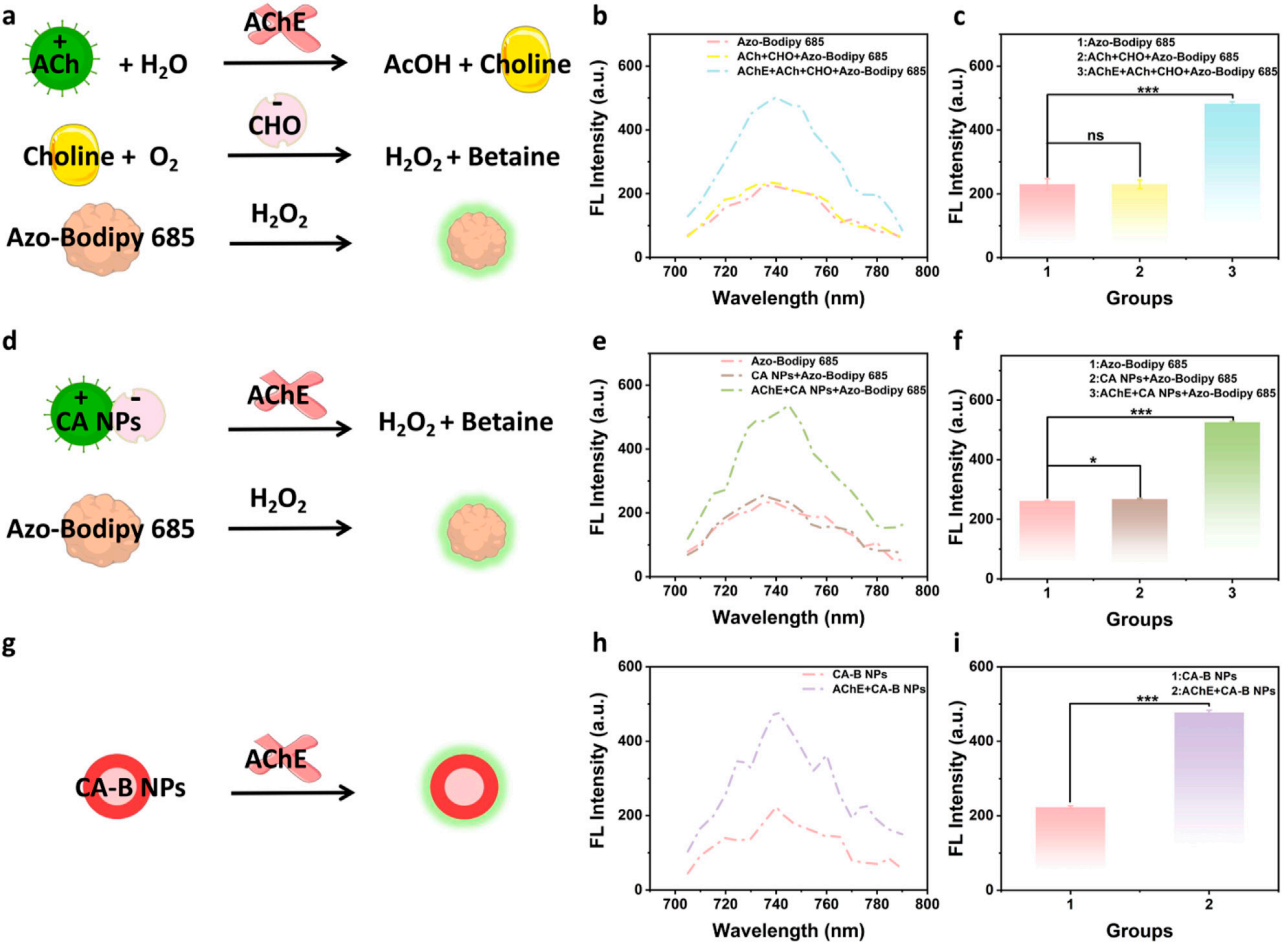
Validation of the CA-B NPs response to AChE. **(a)** Generation of H_2_O_2_ via the cascade reaction chain of the individual components ACh + AChE + CHO; **(b)** Fluorescence spectra and **(c)** maximum fluorescence intensity of H_2_O_2_ verified using Azo-Bodipy 685, data are presented as mean ± SD (n = 3; ns, not significant, ***p < 0.001); **(d)** Generation of H_2_O_2_ via the cascade reaction chain mediated by electrostatically assembled CA NPs + AChE; **(e)** Fluorescence spectra and **(f)** maximum fluorescence intensity of H_2_O_2_ verified using Azo-Bodipy 685, data are presented as mean ± SD (n = 3; *p < 0.05, ***p < 0.001); **(g)** Generation of H_2_O_2_ via the integrated cascade reaction chain of CA-B NPs + AChE; **(h)** Direct verification of fluorescence spectra and **(i)** maximum fluorescence intensity of H_2_O_2_, data are presented as mean ± SD (n = 3; ***p < 0.001). All error bars represent standard deviations based on three parallel measurements.

Based on the varying fluorescence response triggered by Azo-Bodipy 685, proposed and successfully constructed an enzyme cascade reaction sensor composed of AChE, CHO, ACh and Azo-Bodipy 685. At the molecular level, the cascade reaction was first validated: in the primary enzymatic step, the acetylcholine moiety in ACh was hydrolyzed by AChE to produce choline chloride; in the secondary enzymatic step, the resulting choline chloride was further oxidized by CHO to generate H_2_O_2_. The AChE-mediated generation of H_2_O_2_ induced a fluorescence enhancement of Azo-Bodipy 685 (Figure 3a). As shown in Figures 3b,c, significant fluorescence enhancement (ΔF = 252.53 ± 9.08) was observed when Azo-Bodipy 685 was added to a dual-enzyme reaction mixture containing AChE, CHO and ACh. In contrast, no notable fluorescence changes were observed when Azo-Bodipy 685 was added to the single-enzyme reaction mixture (CHO and ACh) or to Azo-Bodipy 685 alone, confirming that Azo-Bodipy 685 specifically responds to the dual-enzyme-mediated cascade reaction (AChE-CHO-H_2_O_2_), thereby enabling the detection of AChE activity.

Building upon this molecular-level validation, we further evaluated the enzyme cascade behavior using CA NPs via fluorescence changes. The schematic diagram was illustrated in Figure 3d. As shown in Figures 3e,f, the addition of AChE and Azo-Bodipy 685 to CA NPs also resulted in significant fluorescence enhancement (ΔF = 257.77 ± 5.27), whereas the mixture of CA NPs and Azo-Bodipy 685 showed negligible difference compared with Azo-Bodipy 685 alone. This result confirms that the electrostatically assembled CA NPs, composed of ACh and CHO, retain full cascade functionality, thereby simplifying experimental procedures while maintaining sensing capability.

Finally, B NPs were encapsulated into CA NPs to form CA-B NPs, thereby further streamlining the operational workflow. As illustrated in Figure 3g, CA-B NPs directly reacted with AChE to yield a pronounced fluorescence enhancement (ΔF = 254.21 ± 2.81), as compared with Azo-Bodipy 685 alone (Figures 3h,i).

In summary, this study developed a charge-driven, self-assembled, all-in-one fluorescence sensor through triple-level optimization of monomer components, electrostatic self-assembly, and all-in-one encapsulation. This system leverages complete component cascade reactions to may alleviate diffusion limitations and steric hindrance, thereby simplifying the experimental procedure, enhancing specificity, and enabling high-throughput detection. This platform holds great promise for rapid determination of AChE activity and OPs content in complex sample matrices.

### 3.4 Evaluation of AChE activity using CA-B NPs and CA NPs

**FIGURE 4:**
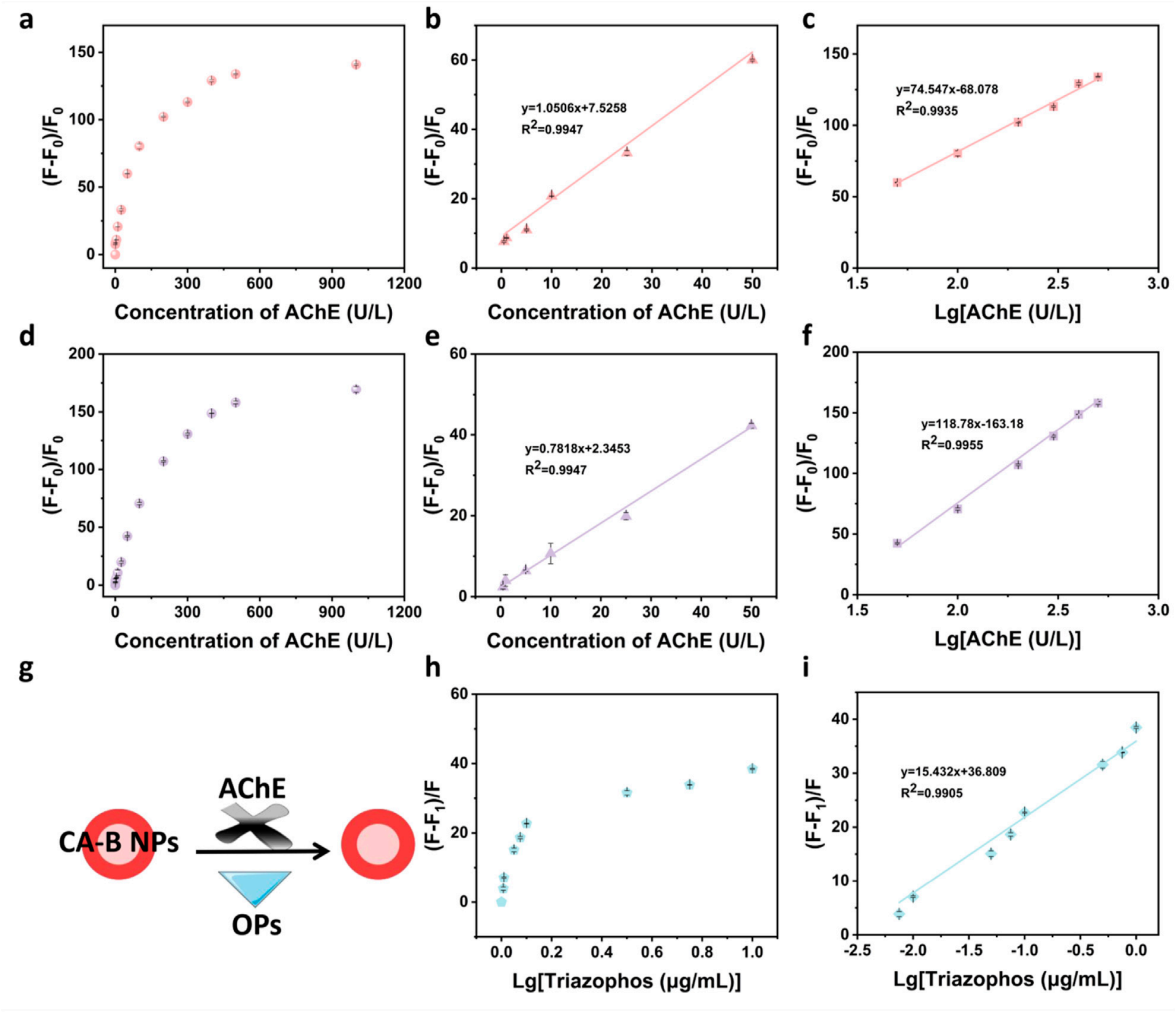
Fluorescence intensity growth rate (F - F_0_)/F_0_ of CA NPs and CA-B NPs for detecting AChE activity and fluorescence inhibition rate (F - F_1_)/F for detecting triazophos. **(a)** Detection of AChE activity (0, 0.5, 1, 5, 10, 25, 50, 100, 200, 300, 400, 500 and 1000 U/L) using CA NPs; Linear relationships between AChE activity [**(b)** 0.5–50 U/L and **(c)** 50–500 U/L] and CA NPs; **(d)** Detection of AChE activity (0, 0.5, 1, 5, 10, 25, 50, 100, 200, 300, 400, 500 and 1000 U/L) using CA-B NPs; Linear relationships between AChE activity [**(e)** 0.5–50 U/L and **(f)** 50–500 U/L] and CA-B NPs; **(g)** Schematic illustration of OPs residue detection via enzyme inhibition using CA-B NPs; **(h)** Detection of triazophos (0, 0.0075, 0.01, 0.05, 0.075, 0.1, 0.5, 0.75 and 1 μg/mL) using CA-B NPs sensor; **(i)** Optimized linear relationship of triazophos detection using CA-B NPs. All error bars represent standard deviations based on three parallel measurements.

The capability of CA NPs to monitor AChE activity was first evaluated. The relative fluorescence response growth rate (GR) (%), defined as GR = (F–F_0_)/F_0_ at 746 nm, exhibited a concentration-dependent increase as AChE concentration increased from 0 to 1000 U/L (Figure 4a). Beyond 500 U/L, however, GR reached a plateau, suggesting that the sensor response saturated and CA NPs achieved their upper detection threshold for AChE activity. Within the AChE concentration range of 0.5–500 U/L, two distinct linear regions were observed: (1) In the range of 0.5–50 U/L, GR showed a good linear correlation with AChE concentration, described by the regression equation: Y = 1.0506X + 7.5258, with a correlation coefficient R^2^ = 0.9947, where X represents AChE concentration (U/L) and Y corresponds to GR (Figure 4b), LOD was calculated to be 0.27 U/L. (2) For the concentration range of 50–500 U/L, a logarithmic transformation of AChE concentration was applied. In this region, a second linear relationship was established: Y = 74.547X – 68.078, with R^2^ = 0.9935, where X = lg [AChE (U/L)] and Y = GR (Figure 4c).

Subsequently, the CA-B NPs system was evaluated for AChE activity detection. The changes in the average fluorescence intensity (Supplementary Figure S5a) and average hydrodynamic particle size (Supplementary Figure S5b) of CA-B NPs were negligible within a week, confirming the stability of CA-B NPs in Tris-HCl buffer solution. In addition, the stability being stored for 1 week under different temperature and humidity conditions were shown in Supplementary Figures S5c-e. Similar dual-linear behaviors were observed within the AChE concentration range of 0.5–500 U/L: (1) In the range of 0.5–50 U/L, a strong linear relationship was obtained, fitted by the equation: Y = 0.7818X + 2.3453, with R^2^ = 0.9947, where X = AChE (U/L) and Y = GR (Figure 4e). The calculated LOD in this system was 0.17 U/L. (2) Within the 50–500 U/L range, logarithmic conversion of AChE concentration again revealed a good linear correlation: Y = 118.78X – 163.18, with R^2^ = 0.9955, where X = lg [AChE (U/L)] and Y = GR (Figure 4f). Importantly, when AChE concentration exceeded 500 U/L and increased up to 1000 U/L, the GR value remained nearly unchanged (Figure 4d), indicating that the fluorescence signal had reached a saturation point. This observation confirms that 500 U/L can be selected as the optimal AChE concentration for the inhibition-based detection of organophosphate pesticides, such as triazophos and chlorpyrifos, ensuring stable and reproducible sensing performance. Besides, the CA-B NPs was compared with the previous works (Supplementary Table S1). From the table, it was observed that the CA-B NPs behaved comparable results (LOD and linear range) with other reported methods, revealing its superior performance in AChE determination.

### 3.5 Detection of triazophos and chlorpyrifos using CA-B NPs and real sample analysis

To demonstrate the enzymes-mediated CA-B NPs in biochemical applications, the method was used to detect triazophos and chlorpyrifos. Under optimized assay conditions, a series of triazophos solutions at varying concentrations were tested using the CA-B NPs. The principle was shown in Figure 4g. As the concentration of triazophos increased, a continuous enhancement in the fluorescence inhibition signal was observed (Figure 4h). When transformed into logarithmic scale, the fluorescence response inhibition rate (IR) (%), defined as IR = (F–F_1_)/F at 746 nm, exhibited a linear relationship with the logarithm of triazophos concentration. The resulting calibration equation was Y = 15.432X + 36.809, with a correlation coefficient R^2^ = 0.9905, where X represented lg [triazophos (μg/mL)] and Y corresponded to IR (Figure 4i). The LOD was determined to be 4.1 ng/mL.

Similarly, chlorpyrifos was evaluated under the same conditions. A comparable linear fluorescence response was obtained (Supplementary Figure S6a), yielding a regression equation Y = 28.582X + 46.518 and R^2^ = 0.9958, where X = lg [chlorpyrifos (μg/mL)] and Y = IR (Supplementary Figure S6b). The corresponding LOD for chlorpyrifos was calculated to be 23.5 ng/mL. These results indicate that the CA-B NPs-based exhibits high sensitivity and a broad dynamic range for the detection of OPs such as triazophos and chlorpyrifos.

To further validate the reliability and practical applicability of the CA-B NPs, real sample analysis was performed using Chenpi, a traditional Chinese medicinal herb. A standard addition approach was employed by spiking triazophos into the sample matrix at concentrations of 0.05, 0.1 and 0.5 μg/mL. The recovery rates for triazophos were found to be approximately 88.13%–113.09%, demonstrating good consistency with the spiked concentrations and confirming the accuracy of the sensor in complex herbal matrices. Moreover, fifteen batches of commercial Chenpi samples were randomly selected for organophosphate residue screening. Pesticide extraction was performed following the sample preparation protocols outlined in the Chinese Pharmacopoeia for pesticide residue determination. As shown in Supplementary Figure S7, triazophos was detected in three of the tested batches, designated as S2410050, S240729 and S2404720. These findings further confirm the feasibility and robustness of the CA-B NPs for practical application in real-world herbal sample monitoring (Ge et al., 2023).

## 4 Conclusion

In this study, a condensation-driven fluorescence sensing strategy was successfully developed for the rapid detection of OPs by constructing an “electrostatic adsorption-driven cascade reaction chain”. Leveraging the electrostatic complementarity ACh and CHO, CA NPs were formed and used to encapsulate the Azo-Bodipy 685, yielding a nanoscale fluorescence sensor CA-B NPs with integrated cascade reactivity and enhanced sensing performance. This sensor system presented three major innovations: (1) Optimization of dual enzyme reaction via spatial confinement effect: By utilizing the electrostatic self-assembly of ACh and CHO, a nanoscale reactive microenvironment was established on the surface of Azo-Bodipy 685. This spatial confinement and co-localization structure significantly shortened the substrate diffusion distance to the nanometer scale and increased the local concentration of choline chloride, which may contribute to improved diffusion limitations and steric hindrance effects caused by heterogeneous system components in conventional sensors. As a result, an optimized “reaction-diffusion equilibrium” was achieved, which greatly enhanced enzymatic efficiency within the confined space. (2) All-in-one cascade signal amplification mechanism: A complete biocatalytic cascade (AChE-CHO-H_2_O_2_) was embedded into the sensor architecture through electrostatically driven self-assembly, enabling a streamlined design that allows *in situ* reaction and signal transduction. This cascade system leveraged the inherent amplification nature of the enzymatic cascade, in which hydrogen peroxide generated by the cascade directly modulates fluorescence intensity. Notably, the fluorescence output was inversely correlated with AChE inhibition caused by OPs, allowing for sensitive and rapid quantification of OPs through a single-step measurement. (3) Practical application and on-site capability: The sensor simplified the detection procedure, enabling one-step OPs detection without the need for complex sample pretreatment. Even within complex matrices such as Chenpi, the system retained a recovery rate of approximately 88.13%–113.09%, demonstrating excellent matrix tolerance and anti-interference capability. Furthermore, the entire detection process could be completed within 20 min, fulfilling the urgent requirement for high-throughput, on-site OPs screening in traditional Chinese herbal medicines cultivation and processing environments. Despite the sensor shows promise in complex matrices, its applicability across a broader spectrum of agricultural products and environmental samples requires further validation. Future work will focus on overcoming these constraints and broadening the platform’s utility. A significant goal is to explore its application beyond pesticide detection, adapting the nanoconfined cascade reaction strategy for the detection of other analytes in various environmental and biomedical fields. Collectively, this work proposes a novel “reaction-diffusion synergy” paradigm that not only overcomes intrinsic mass transport limitations in traditional sensor systems but also provides a generalizable strategy for spatially controlled nanoconfined cascade reactions. This approach may offer valuable insights into the future design of high-efficiency biosensing platforms for pesticide residue analysis and beyond.

## Data Availability

The original contributions presented in the study are included in the article/Supplementary Material, further inquiries can be directed to the corresponding authors.
