# Supplementary material for: Electrostatically driven fluorescent sensor for rapid detection of AChE activity and organophosphate pesticides via dual-enzyme cascade amplification
Source: Front Pharmacol. 2025 Nov 28;16:1679948. doi: 10.3389/fphar.2025.1679948 (PMC12698611; doi:10.3389/fphar.2025.1679948)
Supplement: Supplementary file 1 [file Supplementaryfile1.docx]

Supplementary Material

# Reagents Detail

Chemical structures of Azo-Bodipy 685, ACh, triazophos and clorpyrifos.


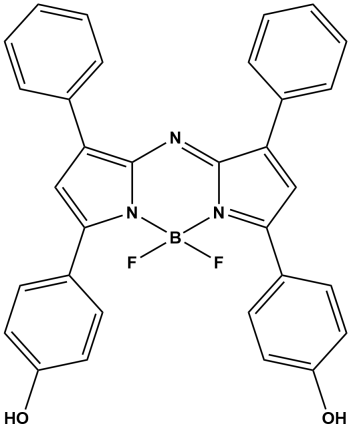


Azo-Bodipy 685


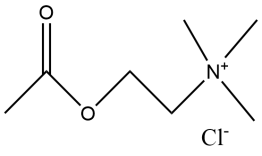


ACh


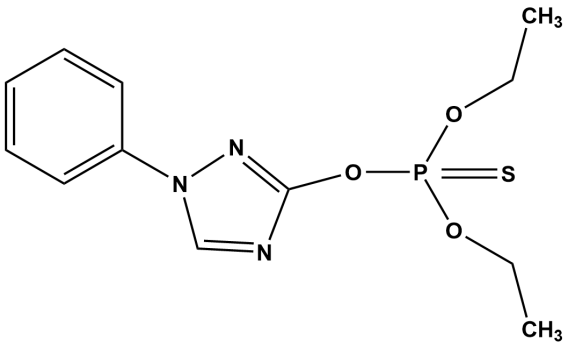


Triazophos


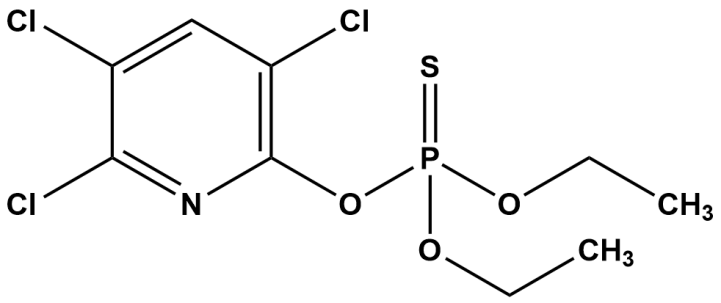


Clorpyrifos

**Supplementary Figure 1.** The structures of Azo-Bodipy 685, ACh, triazophos and clorpyrifos.

# Methods Detail

1. Optimization of the molar ratio of CHO and ACh in CA NPs components

The molar ratio of components (CHO and ACh) in the enzyme cascade amplification reaction system was optimized. First, 80 μL of ACh at different molar amounts (2.5, 5, 10, 15, and 20 μmol) was mixed and stirred with 20 μL of CHO (0.2 U/μL) in Tris-HCl buffer to obtain various CA NPs. Then, AChE solution (5 μL, 0.01 U/μL) was placed into a microcentrifuge tube and incubated at 37°C for 5 min. Subsequently, 40 μL of various CA NPs were continued to be added, mixed thoroughly, and then continued to be incubated for another 20 min at 37°C, and finally, 50 μL of DMSO solution containing Azo-Bodipy 685 was added to terminate the enzyme reaction and to further detect fluorescence signals (λex/em = 706/746 nm).

1. Optimization of stirring reaction time for components in CA NPs (CHO, ACh) and CA-B NPs (Azo-Bodipy 685)

To obtain the optimal process parameters, the stirring time for the synthesis of CHO and ACh components in the CA NPs and the Azo-Bodipy 685 component in the sensor CA-B NPs was optimized. First, 20 μL of CHO solution (0.2 U/μL) was added to 900 μL of Tris-HCl buffer solution (pH = 8) with magnetic stirring (1000  rpm) for 1 h. Sequentially, 80 μL of ACh solution (50 mg/mL) were added, and the mixture was stirred at 1000 rpm for various durations (0.75, 1, 1.5 and 2  h) to obtain different CA NPs. Then, AChE solution (5 μL, 0.01 U/μL) was placed into 40 μL different CA NPs and incubated at 37℃ for 20 min. And finally, 50 μL of DMSO solution containing Azo-Bodipy 685 was added to terminate the enzyme reaction and to further detect fluorescence signals (λex/em = 706/746 nm).

Similarly, 60 μL of Azo-Bodipy 685 fluorescent probe (1 mg/mL in ACN) was slowly added dropwise into 900 μL of Tris-HCl buffer solution, during which the solution was stirred at 1000 rpm for various durations (0.75, 1, 1.5, and 2  h) to yield different fluorescent nanoparticles containing the probe of Azo-Bodipy 685 (B NPs). Subsequently, CHO (20 μL, 0.2 U/μL) and ACh (80 μL, 50 mg/mL) solution were introduced into the system and stirred for an additional 2.5 h. The AChE solution was pre-incubated at 37℃ for 5 min, followed by the addition of 40 μL CA-B NPs. After thorough mixing, the mixture was incubated in the dark at 37℃ for an additional 20 min. The reaction was then terminated by adding 50 μL of DMSO, and the intensity of fluorescence signals were recorded using a microplate reader (λex/em=706/746 nm).

1. The stability of CA-B NPs under different temperature and humidity environmental conditions

The long-term stability of CA-B NPs under different environments was tested. Firstly, CA-B NPs was stored away from light at different temperatures (4, 25 and 37°C), and its stability in Tris-HCl buffer was tested for 1, 3, 5 and 7 days. Figure S5c showed that the average hydrodynamic size changes negligible within one week when stored at 4°C, confirming its stability in the Tris-HCl buffer. At 25°C, the size of the nanoparticles gradually increased over time. Although an increase was observed at a relatively high temperature of 37°C, the size of the nanoparticles remained relatively stable within 7 hours (Figure S5e), and the observed changes were very small, demonstrating good short-term stability. Then, CA-B NPs was stored away from light at different humidity levels (25, 50 and 75%RH), and its stability in Tris-HCl buffer was tested for 1, 3, 5 and 7 days. Figure S5d showd the influence of relative humidity over time on the stability of nanoparticles. At 25%RH, the particle size increased significantly, indicating that aggregation might occur. At 75%RH, the initial size slightly decreased to approximately 150 nm. In contrast, at 50%RH, the size remained approximately at 180 nm within 5 days, with better stability, highlighting the crucial role of medium humidity in maintaining the integrity of nanoparticles. These findings indicated that CA-B NPs was field-operable in different environments.

1. Detection of triazophos in real samples

Fifteen batches of authentic Chenpi samples were selected. First, 5 μL of Tris-HCl buffer solution, methanol solution, methanol extract of the sample was respectively mixed with 5 μL of AChE solution (0.01 U/μL) and incubated at 37℃ for 20 min. Then, 40 μL of CA-B NPs was added, and the mixture was further incubated for 20 min at 37℃. Finally, 50 μL of DMSO was added to terminate the reaction, followed by fluorescence measurements. When the fluorescence detection result was |F_sample_ – F_Tris-HCl_| – |F_methanol_ – F_Tris-HCl_| > 0, it indicated that the sample contained residual triazophos. Each experiment was performed in triplicate for validation. Meanwhile, the samples were analyzed by LC-MS with results summarized in Supplementary Table S2.

# Supplementary Figures





**Supplementary Figure 2.** The response of the B NPs to different volumes (0, 10, 20, 30, 40, 50 μL) of 30% H_2_O_2_. All error bars represent standard deviations based on three parallel measurements.


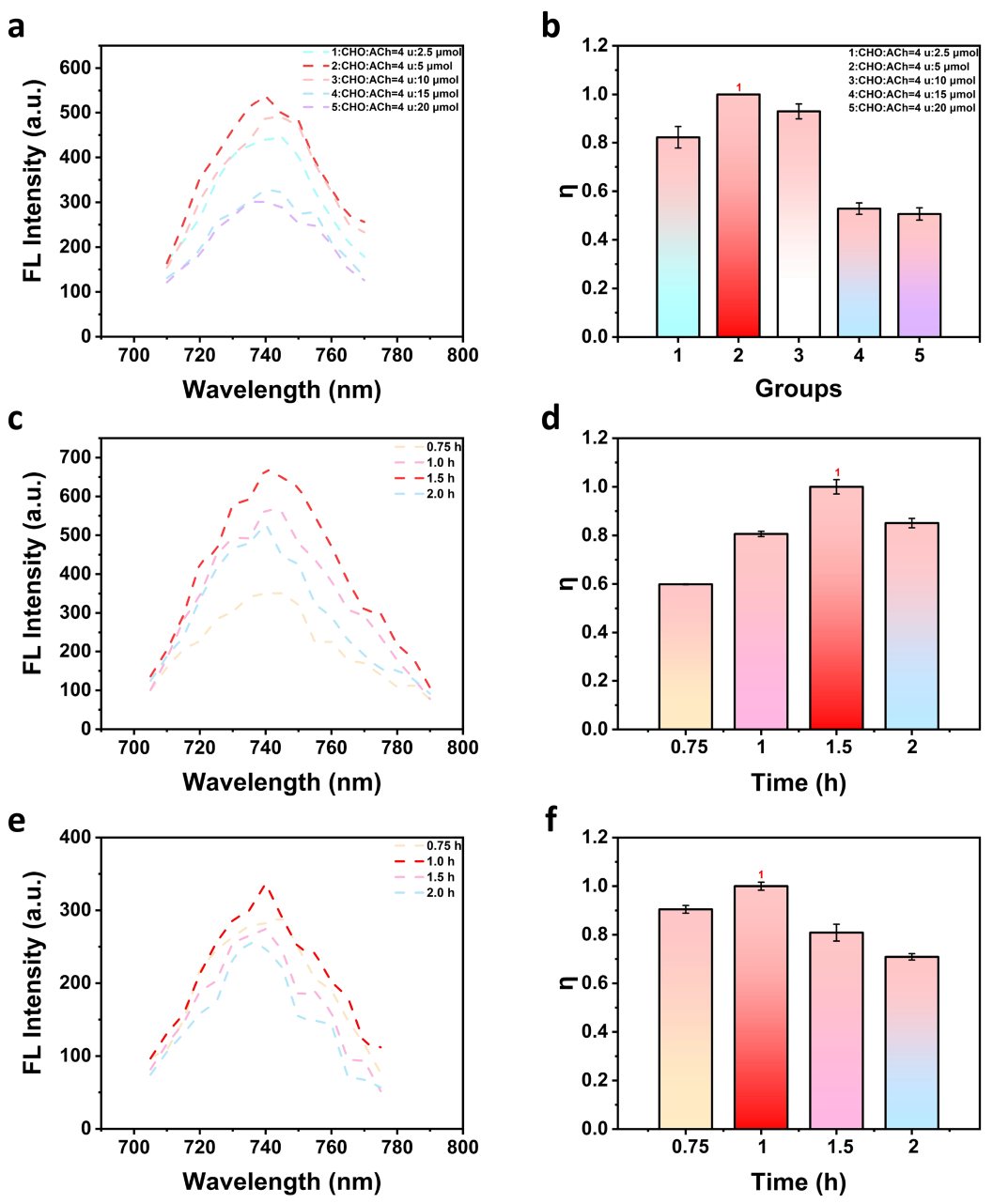


**Supplementary Figure 3.** (a) Fluorescence spectra and (b) intensity of CA NP synthesized at the different molar ratio of CHO and ACh; (c) Fluorescence spectra and (d) intensity of CA NPs (ACh) synthesized at different reaction time; (e) Fluorescence spectra and (f) intensity of B NPs (Azo-Bodipy 685) synthesized at different reaction time. All error bars represent standard deviations based on three parallel measurements.

**
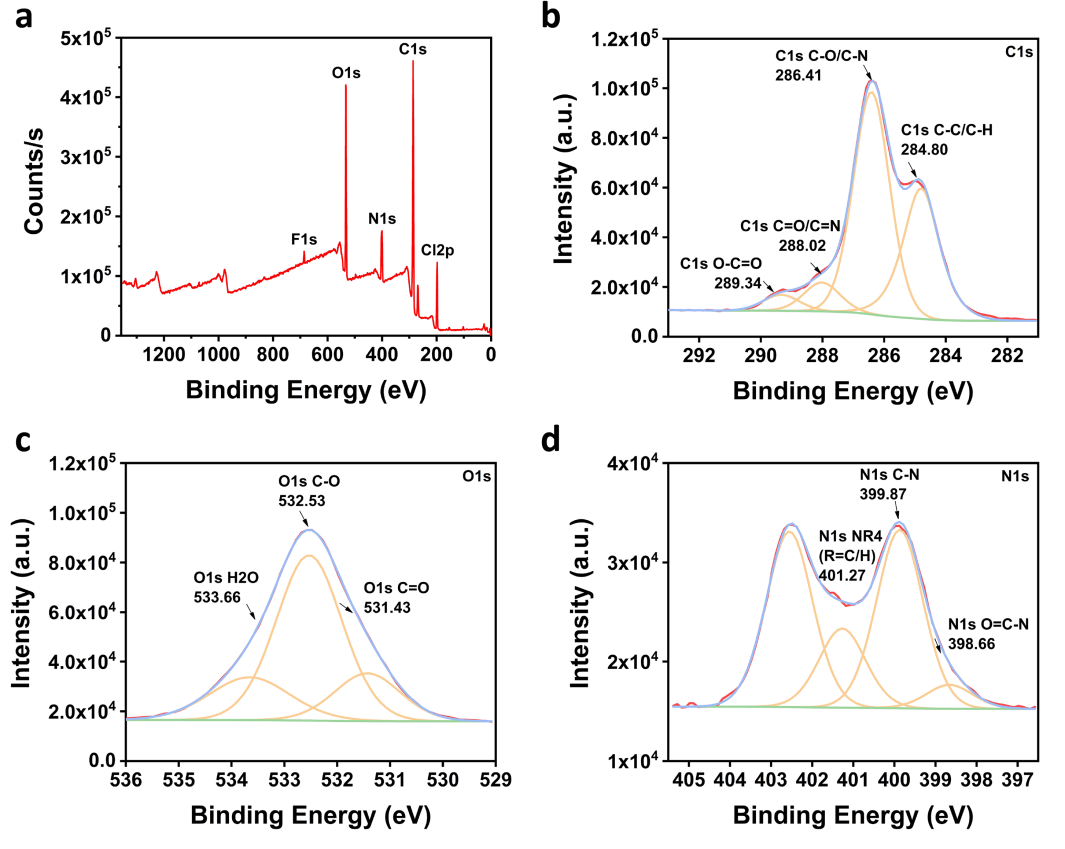
**

**Supplementary Figure 4.** (a) XPS of CA-B NPs; (b) C1s, (c) O1s and (d) N1s of XPS spectra for CA-B NPs.

**
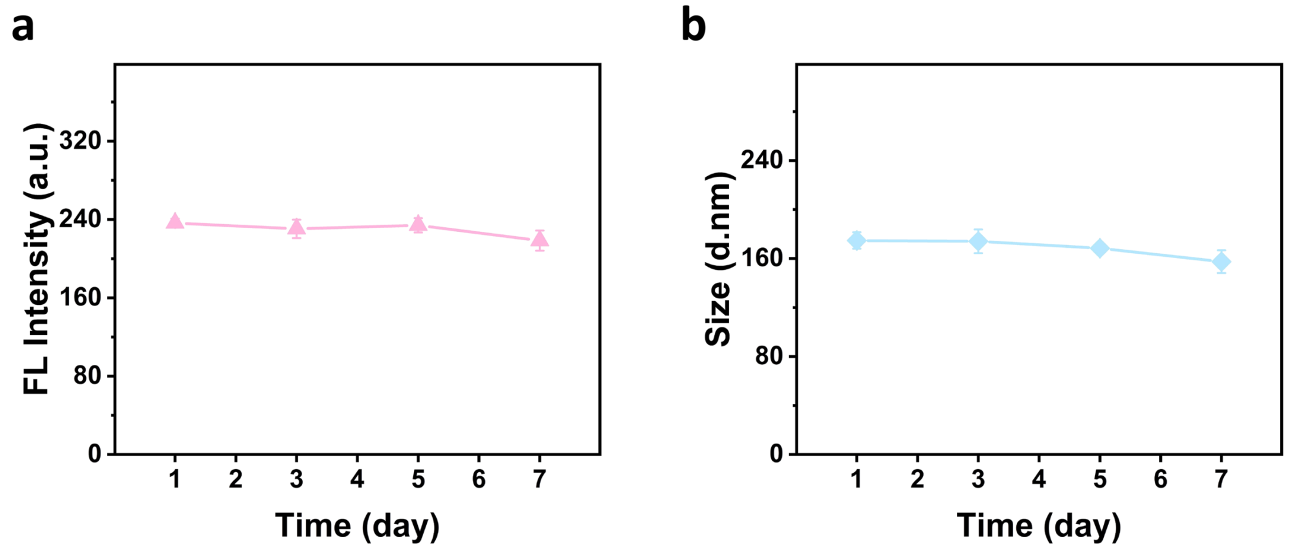
**

**
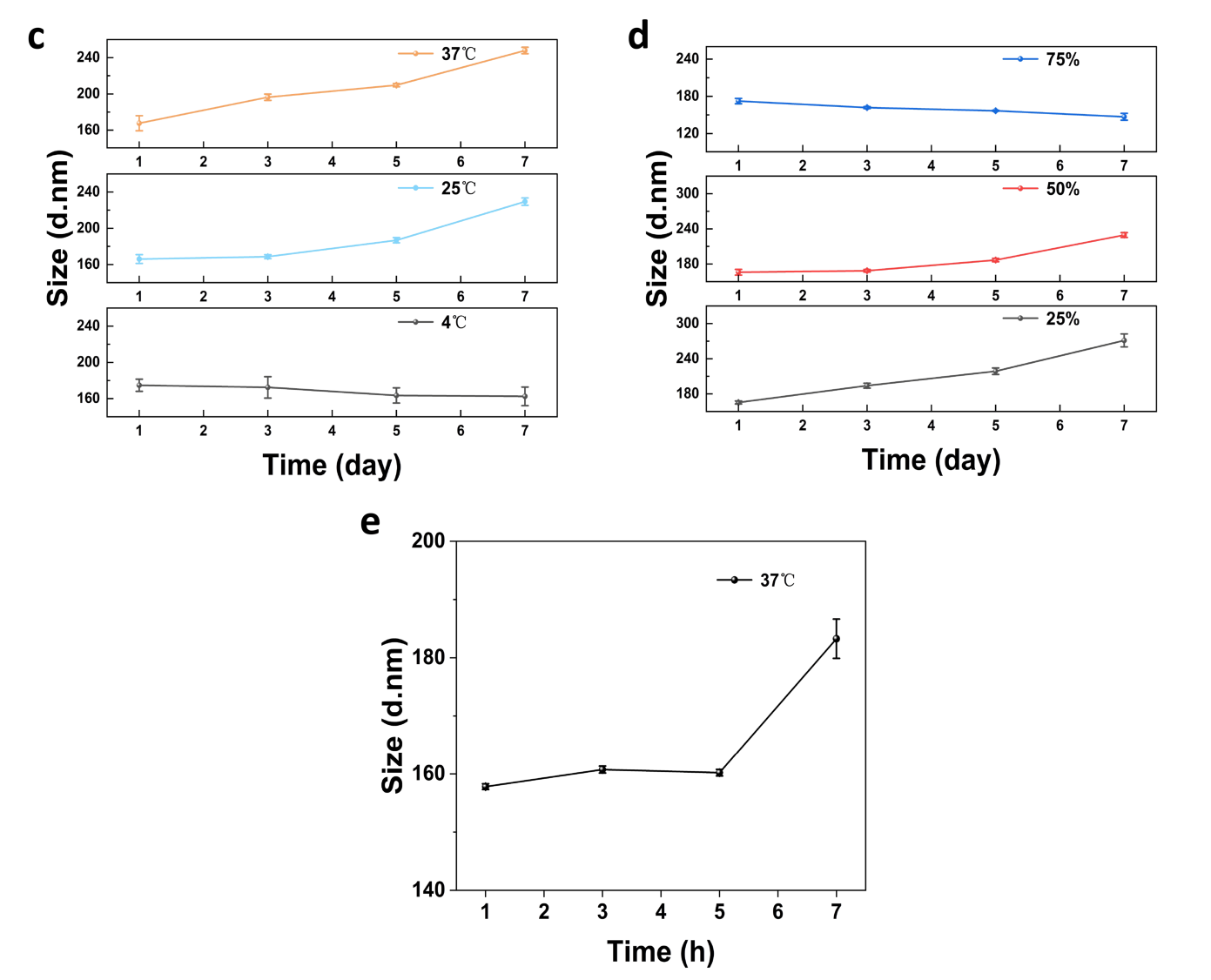
**

**Supplementary Figure 5.** (a) Fluorescence intensity of CA-B NPs during seven days; (b) Hydrodynamic diameter of CA-B NPs during seven days. (c) Hydrodynamic diameter of CA-B NPs at 4℃, 25℃ and 37℃ during seven days. (d) Hydrodynamic diameter of CA-B NPs at 25%RH, 50%RH and 75%RH during seven days. (e) Hydrodynamic diameter of CA-B NPs at 37℃ during seven hours. All error bars represent standard deviations based on three parallel measurements.


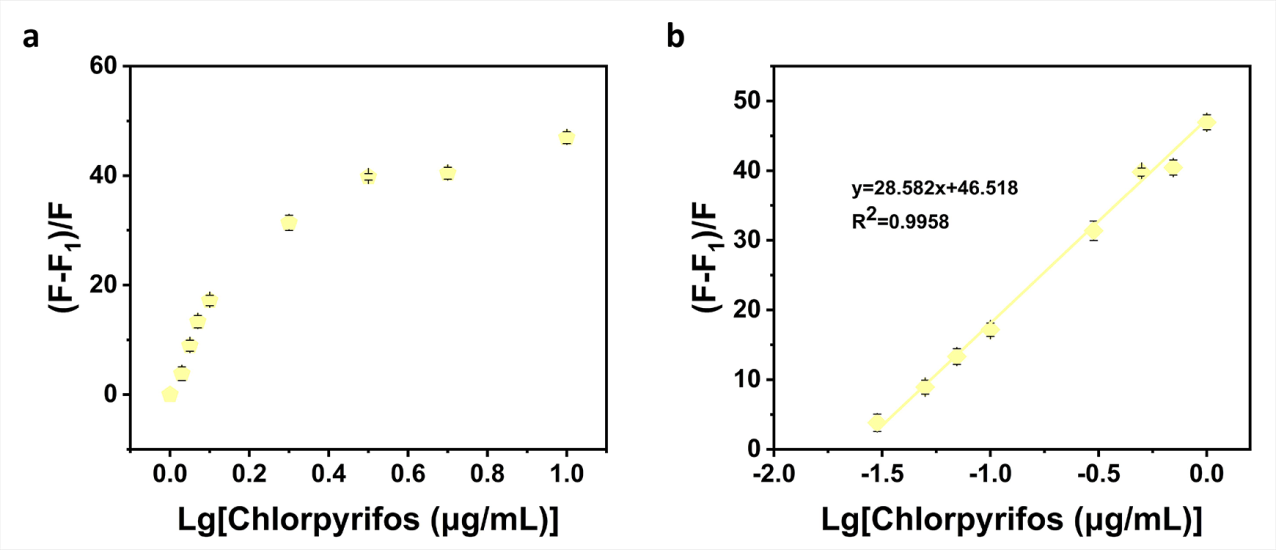


**Supplementary Figure 6.** (a) The figure detection of [clorpyrifos](https://www.chemsrc.com/en/cas/2921-88-2_746254.html) (0, 0.0075, 0.01, 0.05, 0.075, 0.1, 0.5, 0.75, and 1 μg/mL) using CA-B NPs; (b) Optimized linear relationship of [clorpyrifos](https://www.chemsrc.com/en/cas/2921-88-2_746254.html) detection using CA-B NPs. All error bars represent standard deviations based on three parallel measurements.





**Supplementary Figure 7.** The detection of pesticide residues in different samples by CA-B NPs. All error bars represent standard deviations based on three parallel measurements.

**Supplementary Tables**

**Table S1.** Comparison of the CA-B NPs with other reported methods

| **Method** | **LOD (U/L)** | **Linear range (U/L)** |
| --- | --- | --- |
| CDs | 0.1 | 0.2–14 |
| PBMO | 0.03 | 0.08–10 |
| BF_2_-cur-Ben | 31 | 500–7000 |
| NFL-SF | 0.20 | 1–20 |
| Fe/Co-NC | 0.36 | 0.67–6 |
| CA-B NPs | 0.17 | 0.5–1000 |

**Table S2.** Results of the Chenpi samples by the LC-MS

| **Sample Number** | **Pesticide Content (mg/kg)** |
| --- | --- |
| S2410050 | \ |
| S2409650 | \ |
| S2407290 | \ |
| S2406550 | \ |
| S2406090 | \ |
| S2405170 | \ |
| S2404720 | 0.019 |
| S2404830 | \ |
| S2404160 | \ |
| S2403480 | \ |
| S2403340 | \ |
| S2403100 | \ |
| S2402270 | \ |
| S2402010 | \ |
| S2402020 | \ |
